# Prevalence and Phenotype of Lower Urinary Tract Symptoms in Fibromyalgia: A Retrospective Observational Study at a Single Tertiary Medical Center

**DOI:** 10.3390/jcm14155584

**Published:** 2025-08-07

**Authors:** Jackson McClain, Gustavo Capo, Martha Terris, Pablo Santamaria, Noelle A. Rolle

**Affiliations:** 1 The Medical College of Georgia, Augusta University, Augusta, GA 30912, USA; 2 Department of Urology at Wellstar MCG Health, Augusta, GA 30912, USA; 3 Department of Rheumatology at Wellstar MCG Health, Augusta, GA 30912, USA

**Keywords:** fibromyalgia, lower urinary tract symptoms, overactive bladder, stress urinary incontinence, interstitial cystitis, irritable bowel syndrome, generalized anxiety disorder

## Abstract

**Background**: Fibromyalgia syndrome (FMS) is a complex condition with poorly understood pathophysiology, characterized by widespread pain and an increasing recognition of its associations with genitourinary symptoms. The objective of this study was to characterize the prevalence, phenotype, and common comorbidities of lower urinary tract symptoms (LUTS) in women with FMS. **Methods**: A retrospective observational study was conducted using electronic medical records of 440 women diagnosed with FMS at a single institution between 1 January 2018, and 1 January 2024. Study subjects were evaluated for diagnoses associated with LUTS, including interstitial cystitis (IC), overactive bladder (OAB), and stress urinary incontinence (SUI), alongside comorbidities such as irritable bowel syndrome (IBS), generalized anxiety disorder (GAD), and major depressive disorder (MDD). Multivariate analyses were performed to assess predictors of conditions associated with LUTS. **Results**: LUTS were identified in 37.0% of FM patients. GAD and IBS were significantly associated with conditions associated with LUTS (OR = 4.62; OR = 8.53, *p* < 0.001). SUI was present in 17.05% of patients, falling between survey-based and confirmed prevalence rates in the general population. IC was diagnosed in 2.95% of FMS patients. OAB was observed in 6.8% of patients and associated with GAD (OR = 5.98, *p* < 0.001). **Conclusions**: This study highlights a substantial burden of diagnoses associated with LUTS in patients with FMS. There is relatively high prevalence of SUI and IC in this dataset. IBS and GAD were commonly found to co-occur with one or more LUTS-associated condition. Future prospective studies are needed to investigate a multimodal approach to the treatment of LUTS in these patients.

## 1. Introduction

Fibromyalgia Syndrome (FMS) is characterized by widespread pain, stiffness, and soreness primarily in the muscles, classically presenting with “tender points” at various locations of the body. Historically, the prevalence of FMS in the general U.S. population has been estimated at 2–3%, though recent research suggests that this may be an underrepresentation, particularly in the context of the COVID-19 pandemic [1]. Emerging evidence indicates that up to 30–40% of convalescent COVID-19 patients develop chronic widespread pain and fatigue meeting the 2016 diagnostic criteria for FMS, suggesting a potential rise in population burden following the pandemic [2,3]. Most commonly, FMS is diagnosed in Caucasian women between the ages of 20 and 55. The pathophysiology of FMS is complex, with multiple proposed mechanisms that have varying levels of evidence for each. While altered central pain processing—including enhanced sensory input and reduced pain inhibition—remains a leading theory, other contributing mechanisms include autonomic nervous system dysfunction, neuroinflammation, genetic susceptibility, hypothalamic–pituitary–adrenal axis dysregulation, and psychosocial stressors [4,5,6]. Genetic predisposition, autonomic system perturbations, and stress are likely contributory, yet their exact role remains largely undefined [4]. In recent years, we have come to understand more about this complex interplay and its frequent comorbid conditions—migraines, obstructive sleep apnea, generalized anxiety disorder, major depressive disorder, irritable bowel syndrome (IBS), and others—a complex polysymptomatology that is labor-intensive and challenging to treat clinically.

The term “lower urinary tract symptom” (LUTS) was coined in 1994 by Paul Abrams to create a general term for irritative voiding symptoms, a broad spectrum of disease, which was not shoehorned into a mold made for a single organ such as the prostate [7]. LUTS is a broad term by necessity, as the binary distinction between “failure to store” and “failure to empty” fails to address that many diseases with LUTS phenotype do not always fit neatly into one or the other [8]. LUTS are increasingly common in our aging population, and often underreported by patients [9]. LUTS may result in substantial morbidity, including sleep disruption, impaired quality of life, and mental health decline, and are frequently associated with systemic inflammatory conditions [10]. While there is growing interest in examining the overlap between FMS and LUTS, the nature of this relationship is yet to be clearly defined.

Several previous works have identified a link between fibromyalgia and a variety of irritative voiding symptoms. A 2022 meta-analysis of primarily observational studies noted a connection between overactive bladder (OAB) and FMS, but few large scale case–control studies have been carried out on the subject [11,12]. OAB is just one LUTS etiology among many. Emerging evidence suggesting a clinical—and potentially pathophysiological—association between FMS and interstitial cystitis, stress incontinence, and others have been published in recent years [13,14,15].

Few studies have evaluated the prevalence and distribution of LUTS subtypes in patients with fibromyalgia or systematically assessed the impact of FMS-associated comorbidities on LUTS burden. Historically viewed as a musculoskeletal pain disorder, FMS has only recently been recognized as a multisystem condition linked to genitourinary, gastrointestinal, and neuropsychiatric symptoms [16,17]. IBS is frequently comorbid in patients with FMS. One study reported that patients with IBS had a prevalence odds ratio of 1.8 with respect to co-occurrence of fibromyalgia, when compared to a cohort without IBS [18]. Previous retrospective studies have reported an association between IBS and various LUTS, including urinary frequency, nocturia, and urinary urgency scores among patients with FMS [19]. Both depression and anxiety have been found to correlate with LUTS—particularly those symptoms found in overactive bladder, such as urinary urgency and frequency—both in the general population and in patients with FMS. Autoimmune disorders are common among the FMS patient population and possess a suggested relationship to irritative LUTS independently, although this association has not been studied in patients with FMS [20,21,22]. This study aims to fill in this gap in understanding by characterizing the prevalence, phenotype, and common comorbidities associated with LUTS in patients with FM.

## 2. Materials and Methods

### 2.1. Study Design and Setting

We conducted a retrospective observational cohort study at a tertiary academic medical center in the southeastern United States. This study was approved as exempt by the institutional review board (IRB) at the institution.

### 2.2. Data Sources and Data Collection

In this retrospective observational study, we obtained medical records for 440 women with a record of an outpatient rheumatology visit and diagnosis of fibromyalgia between 1 January 2018 and 1 January 2024, at a tertiary care facility. All data were de-identified prior to analysis, and informed consent was waived by the IRB under Exemption Category #4C.

### 2.3. Inclusion and Exclusion Criteria

Inclusion criteria: All patients must be females aged 18 years or older at the time of their first visit with the rheumatologist and have a recorded diagnosis of fibromyalgia as defined in the 2016 Revisions to the 2010/2011 Fibromyalgia Diagnostic Criteria. Only patients who had received a diagnosis of fibromyalgia from a board-certified rheumatologist were included, which aimed to reduce the number of potential false positive cases referred from general practitioners. To ensure that a complete medical history was available for each patient, this study only included patients who had a viewable history and physical note from their primary care provider, either as a referral document from an outside facility or from a provider within the institution.

**Figure 1:**
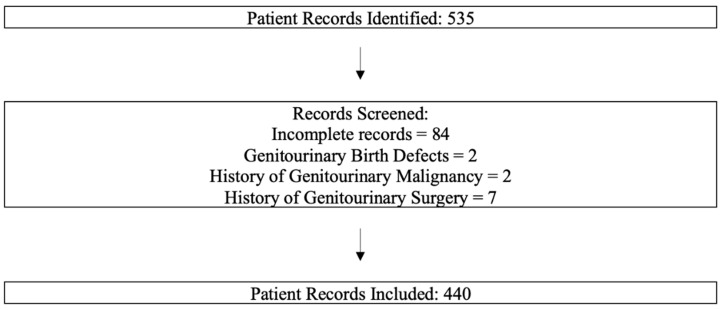
STROBE Flowchart: This flowchart outlines our patient identification and inclusion methods.

Exclusion criteria: Patients with several conditions were excluded from the study (Figure 1):

(a) Genitourinary birth defects of the kidney, ureter, or bladder;
(b) Prior or current genitourinary malignancy;
(c) A history of genitourinary surgery during the study period.

These exclusions were applied to reduce the potential confounding effect of urinary symptoms caused by structural or oncologic processes unrelated to FM.

### 2.4. Collection of Relevant Variables

The medical record numbers, FM status, initial rheumatology visit date, age, and sex were obtained using an IRB-approved data mining tool (Power Trials). This initial dataset was stored in a virtual access box secured by 256-bit AES encryption. Patient charts were then examined for the diagnoses of interest to this analysis The LUTS diagnoses recorded in our study included: chronic pelvic pain or chronic pelvic pain syndrome, interstitial cystitis, recurrent cystitis, overactive bladder, urge incontinence, stress incontinence, mixed incontinence, and nocturia. Several comorbid conditions were also recorded, including history of tobacco use, diagnosis of other autoimmune disorders, irritable bowel syndrome, hypertension, anxiety, and depression.

### 2.5. Statistical Analysis

Statistical analysis was performed using Python version 3.12, available at https://www.python.org/downloads/ (accessed 31 October 2024). The primary endpoint of the study was to characterize the prevalence and phenotype of LUTS in patients with fibromyalgia. The secondary endpoint of this study was to determine the relationship between LUTS and several conditions which are commonly comorbid with fibromyalgia.

Demographic data for age, race, and LUTS subtype within the dataset was reported. Univariate associations between each predictor and the binary outcome of any LUTS diagnosis were evaluated using logistic regression for continuous variables and chi-square or Fisher’s exact tests for categorical variables. Multiple comparisons were controlled with the Benjamini–Hochberg false-discovery rate procedure (q = 0.05). Multivariate analysis was performed to determine which variables remained significant when considering age, race, and comorbidities. Pairwise deletion was used for missing data (<5% of variables) Effect estimates are reported with odds ratios (ORs), 95% confidence intervals, and adjusted *p*-values.

## 3. Results

### 3.1. Notable Findings

Our final cohort consisted of 440 female patients with a diagnosis of FM, following the exclusion of 95 records due to factors such as incomplete documentation, history of genitourinary malignancy, or genitourinary congenital anomalies (Figure 1). The median age was 51.8 years, with a wide distribution ranging from 21 to 87 years, reflecting the broad age spectrum affected by FM (Table 1). The racial composition was nearly evenly split between White (47.0%) and Black (45.2%) participants, aligning with the diverse demographic served by our clinic population.

Among this sample, 163 patients (37.0%) had documentation of at least one lower urinary tract symptom (LUTS). The most commonly recorded diagnosis was SUI at 17.0%, followed closely by urge incontinence (UI) at 15.2%, and mixed incontinence (MI) at 10.9%. Chronic pelvic pain syndrome (CPPS) was also frequently reported (13.6%), suggesting a notable overlap between pain syndromes and urologic symptoms in this population (Table 2). Other LUTS conditions, including overactive bladder, recurrent cystitis, and urinary frequency, occurred at lower frequencies but further underscore the heterogeneity of urologic complaints in patients with FM.

Beyond LUTS, the cohort exhibited a high prevalence of comorbidities. Obesity or severe obesity was present in 68.0% of the sample. Mental health comorbidities were also prominent, with depression affecting 42.3% of patients and anxiety affecting 34.7%.

### 3.2. Multivariate Analyses

Our multivariate logistic regression (Table 3) identified several key predictors of LUTS among FM patients. Notably, irritable bowel syndrome (IBS) emerged as the strongest independent predictor, with an odds ratio (OR) of 8.53 (95% CI: 4.64–15.71, Bonferroni-adjusted *p* < 0.001). Anxiety also demonstrated a significant association, with an OR of 4.62 (95% CI: 2.74–7.77, *p* < 0.001), reinforcing the important interplay between psychological distress and urologic symptoms. While depression approached significance (OR: 1.57, *p* = 0.0855), it did not meet the Bonferroni-adjusted threshold, suggesting a potential but less robust relationship compared to anxiety.

Contrary to expectations, obesity, age, race, and cardiovascular/metabolic comorbidities (HTN/HLD/CAD) were not significantly associated with the presence of any LUTS in adjusted models. This may reflect the multifactorial and predominantly neuropsychological nature of LUTS in this particular cohort.

### 3.3. Symptom Burden

To assess the breadth of LUTS involvement, a negative binomial regression model (Table 4) was applied to estimate the number of distinct LUTS subtypes per patient. Once again, IBS (IRR: 2.86) and GAD (IRR: 2.23) were significantly associated with higher LUTS symptom burden, defined as the total number of distinct LUTS diagnoses per individual. This suggests that these conditions not only increase the likelihood of any LUTS but may also predispose patients to multiple concurrent urologic complaints, highlighting the clinical complexity and management challenges in this population.

Interestingly, patients with race marked as “Other/Unknown” had a significantly higher LUTS burden (IRR: 2.56, *p* = 0.0479), though the small sample size of this subgroup warrants cautious interpretation.

## 4. Discussion

The present study provides a single-center analysis of LUTS prevalence and associated risk factors in a cohort of 440 women with FM. Of the study participants, 37.0% had one or more LUTS diagnoses. Two LUTS phenotypes in particular—stress urinary incontinence (SUI) and IC—were more prevalent in our dataset when compared to the reported data in the general female population [23,24]. Precise current estimates for the prevalence of these conditions varies based on the methodology used to collect the data, particularly when comparing symptom surveys to studies which utilize administrative coding [25,26]. These findings support the hypothesis that there may be some link between IC and fibromyalgia, although long term observational studies are needed to exclude the possibility of overdiagnosis secondary to increased healthcare utilization in the fibromyalgia patient population. The prevalence of SUI in our dataset falls between the survey-based and ICD-confirmed rates in the general population, although this finding should be interpreted with caution given the high incidence of obesity and severe obesity in our study.

Multivariate analysis demonstrated robust independent associations between the presence of LUTS and both IBS (OR 8.53) and anxiety (OR 4.62) when controlling for other comorbidities and demographic variables. Earlier population-level studies have shown that anxiety is associated with OAB and urinary incontinence [20,21,27]. Psychosocial factors are known to influence bladder function, potentially through alterations in neurotransmitter levels and increased muscle tension [22,28]. While a well-accepted pathophysiologic explanation is not available, the clinically observable correlation between anxiety and LUTS in this patient population suggests a potential role for future studies to investigate the effect of anxiety treatment on LUTS in these patients. If found to be efficacious, this may help to reduce the financial burden of healthcare in patients with fibromyalgia [29]. IBS has previously been shown to frequently co-occur in interstitial cystitis and OAB, which may be explained by dysregulation of the autonomic nervous system and visceral hypersensitivity common in both conditions [20,22]. These hypotheses should be explored in prospective clinical trials to provide improved insight into novel therapeutic approaches to effectively treat these conditions.

This study has several limitations. This single-center study with only a moderate sample size may not represent generalizable findings when considering the diverse population of patients diagnosed with FM worldwide. Furthermore, men were excluded from this study to allow for a more focused assessment of a female patient population without introducing additional sex related factors. Pregnancy status was not controlled for in this analysis, which should be considered when interpreting the findings, given that the prevalence of genitourinary conditions is higher during and after pregnancy. Patient history of obesity was included in this analysis as a binary variable, which limits analysis regarding the magnitude of obesity and its role on LUTS in this population. As with any retrospective chart review, these results should be interpreted carefully since this study was not randomized. Manual keyword search was used to identify LUTS-associated diagnoses and the comorbid conditions; while more sensitive than ICD codes, manually screening patient records introduces misclassification bias into the data collection process particularly when performed without a blinded double-review by a second researcher.

Another key limitation of this study is the potential underdiagnosis of IC within the FMS population. While IC was diagnosed in approximately 3% of our study subjects, this reflects only those with a documented diagnosis and might not capture true prevalence. Prior research has shown that IC can be missed is than 60% of cases [30]. This highlights an ongoing uncertainty and inconsistency in diagnostic practices. Furthermore, the dataset used did not account for emerging conditions such as long COVID which has been associated with chronic widespread pain, fatigue, and OAB which can resemble IC [2,31,32]. Inclusion of long COVID as a comorbidity could enhance understanding of both symptom overlap and disease burden in this cohort but was beyond the scope of our analysis.

## 5. Conclusions

In conclusion, this study reports the prevalence of, and diagnoses underlying the presence of LUTS among patients with fibromyalgia at a single center. Within this patient population, patients with a diagnosis of anxiety or IBS were more likely to have at least one of the LUTS-associated diagnoses. These findings, while preliminary, encourage future prospective studies to investigate targeted clinical interventions to identify and treat LUTS in this patient population.

## Figures and Tables

**Table 1:** Summary of Patient Demographics. This table presents the descriptive statistics of the study population, including age distribution, sex, racial demographics, and the presence comorbidities.

Characteristic	Subcategory	Value
Total Sample	-	n = 440
Age (years)	Mean ± SD	51.8 ± 12.8
	Median (IQR)	52 (44–60)
	Range	21–87
Sex	Female	440 (100.0%)
Race/Ethnicity	White	207 (47.0%)
	Black/African Am	199 (45.2%)
	Hispanic/Latino	12 (2.7%)
	Mixed/Other	10 (2.3%)
	Unknown	8 (1.8%)
	Asian	3 (0.7%)
	Other	1 (0.2%)
Age Groups	<40 years	84 (19.1%)
	40–49 years	109 (24.8%)
	50–59 years	140 (31.8%)
	≥60 years	107 (24.3%)
Comorbidity Burden	Mean ± SD	2.87 ± 1.63
	Median (IQR)	3 (2–4)
	Range	0–7

**Table 2:** Prevalence of LUTS and Other Conditions. This table summarizes the prevalence of individual LUTS diagnoses as well as other conditions found among our study subjects.

Variable	Category/Measure	Percentage
LUTS Conditions	CPPS	13.6%
	Stress Incontinence	17%
	Overflow Incontinence	1.0%
	Mixed Incontinence	10.9%
	Urge Incontinence	15.2%
	Interstitial Cystitis	3.0%
	Recurrent Cystitis	6.4%
	Overactive Bladder	6.8%
	Urinary Urgency	2.5%
	Urinary Frequency	3.6%
	Nocturia	2.5%
	Dysuria	2.0%
	Any LUTS Condition	37.0%
Other Conditions	Obesity	68.0%
	GAD	34.7%
	MDD	42.3%

**Table 3:** Multivariate analysis. This table presents the results of the multivariate analysis examining factors associated with the presence of any LUTS. Odds ratios (OR), 95% confidence intervals (CI), *p*-values, and 5% False Discovery Rate adjusted *p*-values are reported. Statistically significant associations indicate potential risk factors for LUTS in the patient population.

Variable	Adjusted OR (95% CI)	*p*-Value
Age	1.02 (1.00–1.04)	0.1262
Asian	0.00 (N/A)	1.0000
Black	0.69 (0.41–1.14)	0.1472
Hispanic	0.74 (0.16–3.40)	0.6993
Mixed	0.88 (0.17–4.71)	0.8856
Other Race/Unknown	1.34 (0.28–6.34)	0.7125
Tobacco Use	1.12 (0.66–1.91)	0.6764
IBS	8.53 (4.64–15.71)	<0.0001
HTN/HLD/CAD	1.30 (0.76–2.24)	0.3427
Obesity	1.45 (0.82–2.54)	0.1995
MDD	1.57 (0.94–2.64)	0.0855
GAD	4.62 (2.74–7.77)	<0.0001

**Table 4:** Negative Binomial Regression Analysis: Negative Binomial regression was performed to model the count of distinct LUTS subtypes (0–8) per patient. This approach captures symptom “burden” rather than just presence/absence, providing insights into factors associated with symptom multiplicity. Incidence Rate Ratios (IRR) represent the expected multiplicative change in LUTS symptom count for each unit increase in the predictor. An IRR > 1 indicates increased symptom burden.

Variable	IRR (95% CI)	*p*-Value
Asian	0.00 (0.00–inf)	0.9991
Black	0.92 (0.65–1.30)	0.6410
Hispanic	0.97 (0.33–2.88)	0.9604
Mixed	1.89 (0.76–4.69)	0.1711
Other/Race Unknown	2.56 (1.01–6.50)	0.0479
Age	1.01 (1.00–1.02)	0.1688
Tobacco Use	1.22 (0.86–1.72)	0.2676
Other Autoimmune Disease	1.27 (0.92–1.75)	0.1415
IBS	2.86 (2.03–4.04)	<0.0001
IBD	1.92 (0.80–4.62)	0.1449
HTN/HLD/CAD	1.17 (0.81–1.69)	0.4031
Obesity	1.34 (0.91–1.98)	0.1413
MDD	1.24 (0.88–1.76)	0.2188
GAD	2.23 (1.57–3.15)	<0.0001

## Data Availability

The data presented in this study are available on request from the corresponding authors due to institutional privacy policies and restrictions related to protected health information (PHI).

## Abbreviations

BMI	Body Mass Index
CAD	Coronary Artery Disease
CI	Confidence Interval
CPPS	Chronic Pelvic Pain Syndrome
EMR	Electronic Medical Record
FM	Fibromyalgia
HLD	Hyperlipidemia
HTN	Hypertension
IBD	Inflammatory Bowel Disease
IBS	Irritable Bowel Syndrome
IC	Interstitial Cystitis
IQR	Interquartile Range
IRB	Institutional Review Board
IRR	Incidence Rate Ratio
LUTS	Lower Urinary Tract Symptoms
MI	Mixed Incontinence
OAB	Overactive Bladder
OR	Odds Ratio
PHI	Protected Health Information
SD	Standard Deviation
SUI	Stress Urinary Incontinence
UI	Urinary Incontinence
